# Carbon-Based Electrodes for Supercapacitors, with a Focus on Carbon Nanotubes—A Brief Overview

**DOI:** 10.3390/ma18225215

**Published:** 2025-11-18

**Authors:** Lilla Nánai, Klara Hernadi

**Affiliations:** Institute of Physical Metallurgy, Metal Forming and Nanotechnology, University of Miskolc, 3515 Miskolc, Hungary

**Keywords:** energy storage, supercapacitor, vertically aligned carbon nanotubes, electrode materials

## Abstract

Increased needs arising from efficient utilization of renewable energy sources and the emerging use of portable electronic devices have introduced new requirements and challenges, such as fast charging and discharging, high-speed energy delivery, longer lifetime, and recyclability. To meet these demands, the innovative use of supercapacitors is essential, as they can complement the batteries currently in use. One of the major disadvantages of supercapacitors is that their energy storage capacity (5–20 Wh/kg) is currently insufficient, compared to the capacity of batteries (~1000 Wh/kg). Supercapacitors have higher specific power (10 kW/kg) but lower specific energy density, which is another significant disadvantage compared to batteries. This has prompted researchers around the world to find innovative solutions to enhance the energy density of these materials. Carbon-based nanomaterials are one of the most widely used electrode materials for supercapacitors; therefore, the development of carbon-based nanomaterials plays crucial role in evolution of supercapacitors, due to their high electrical conductivity, large specific surface area, and excellent mechanical strength compared to conventional electrode materials graphite, copper, platinum, etc. Significant results have been reported in the scientific literature on novel carbon-based nanostructured materials such as carbon nanotubes, vertically aligned carbon nanotubes, graphene, activated carbon, or carbon nanoballs, which have a hierarchical pore structure, as well as hybrid systems combining these materials and the introduction of alternative electrolytes. This manuscript reviews briefly the background and fundamental characteristics of supercapacitors, classifying them. It also mentions the general electrochemical measurement methods used to evaluate the energy storage properties of supercapacitors, with emphasis on their specific characteristics and limitations. The integral components of supercapacitors, especially electrode materials, are considered to have considerable impact on the performance of supercapacitor devices (e.g., long life cycle, storage capacity, and high power density).

## 1. Introduction

As technology and economy evolve, the global demand for energy keeps growing. Efficient energy storage and conversion are the foundation of today’s society, driven by the mutual promotion of environmental awareness and economic development. Supplies of fossil fuel are limited, and their use causes significant environmental pollution [1,2,3,4]. Therefore, considerable attention has been given to the applicability and integration of renewable solar and wind energy sources into everyday life. Energy utilization and transformation are essential for renewable energy sources, given their global prevalence. Due to low energy density, batteries are not suitable in the long term for reducing the imbalance between renewable energy production and efficient energy storage [5]. The aim of research and development is to lower and step-by-step exclude environmental and economic disadvantages associated with the use of traditional energy sources. The energy generated by renewable energy sources is often unstable, and its direct application faces various obstacles, including solar panels, can only convert sunlight into electricity when sunlight is available. The mechanism of energy storage is not well developed, particularly for seasonal energy (wind and tidal) facing the same challenges. Certain renewable energies are capable of generating electricity continuously; however, these power plants are site-limited due to their requirements. Renewable energy sources are not aligned with peak electricity consumption periods, leading to major energy losses in our daily lives [5]. Thus, energy storage devices are essential for the efficient use of these energies. The technology of energy storage is crucial in managing the variable nature of renewable energies and to satisfy the energy demands of emerging electronic devices [6]. Battery-, fuel cell-, and electrochemical capacitor (also known as supercapacitor (SC))-based electrochemical energy has a key role in meeting today’s energy demands efficiently. While these devices have similar electrochemical properties, the mechanisms of energy storage and conversion differ [7,8,9]. Nowadays, SCs have attracted a great deal of interest owing to their unique characteristics, such as high specific capacity, fast charging and discharging capability, and long lifetime [10]. SCs are capable of delivering high specific power (10,000 W/kg) and short-duration (between seconds and minutes) high current pulses [11]. Their outstanding lifetime, which often extends beyond thousands or even millions of charge and discharge cycles, sets them apart from traditional batteries (Table 1). Such exceptional robustness results due to the electrostatic character of energy storage in supercapacitors, which minimizes quality degradation through multiple cycles [12]. SCs can operate by standing alone or integrated with other storage systems such as batteries and can be used in a variety of applications including industrial power management, solar energy production, hybrid electric vehicles, and consumer electronics [13,14]. In addition, their recyclability and longer lifetime meet the demands of environmental sustainability.

Despite many advantages of SCs, the principal weakness of SCs is their relatively low energy density (5–20 Wh/kg), which is approximately 20–40 times lower than the conventional rechargeable Li-ion batteries (100–265 Wh/kg) [14,15].

**Table 1:** Comparison of properties of commercial supercapacitors and lithium-ion batteries.

Parameters	Electric Double-Layer Supercapacitor	Lithium-Ion Battery	Refs.
Charge capacity	1–5000 F/3.04 Wh	1000–100,000 F/12.1 Wh	[16,17]
Energy density [Wh/kg]	1–20	20–300	[14,15,16,17]
Power density [W/kg]	2000–10,000	50–200	[11,16]
Maximum power [W]	7020	18	[16]
Charge time [s]	1–60	3600–18,000	[16]
Self-discharge	5–60% per two weeks	<4% per month	[18,19]
Nominal voltage [V]	2.7–3.0	3.6	[16,17]
Cycle life (charge–discharge cycles)	500,000–1,000,000	250–1000	[20,21]
Temperature range [°C]	−40 to 70	−20 to 60	[22,23]
Price per kWh [US-$]	5000–10,000	100–1000	[16,24]

Hence, extensive research has aimed to improve the energy storage capacity of SCs while maintaining their high power output capability. The challenge is to design versatile and improved energy storage systems for a wide range of applications. To achieve this aim, researchers focus on improving energy density, with particular emphasis on electrode material and structure innovation [25,26,27]. Recent studies have focused on a particular group of materials, offering an overview of recent developments in the state of the art, the most characteristic physicochemical properties, supercapacitive performance, and methods of fabrication of such materials [28,29,30].

## 2. Supercapacitors—A Brief History

The concept of electrical charge storage on surfaces can be traced back to the days of ancient Greece, where the concept was inspired by the friction properties of amber [31]. The molecular understanding of electricity can be traced back to the 19th century, to the early studies on electrons by Michael Faraday and later by J. J. Thomson and Millikan [32]. The Leyden jar, invented by Pieter van Musschenbroek, marked a crucial step forward, introducing the principles of charge separation and storage, which was originally known as a “condenser” and later as a “capacitor” [33].

**Figure 1:**
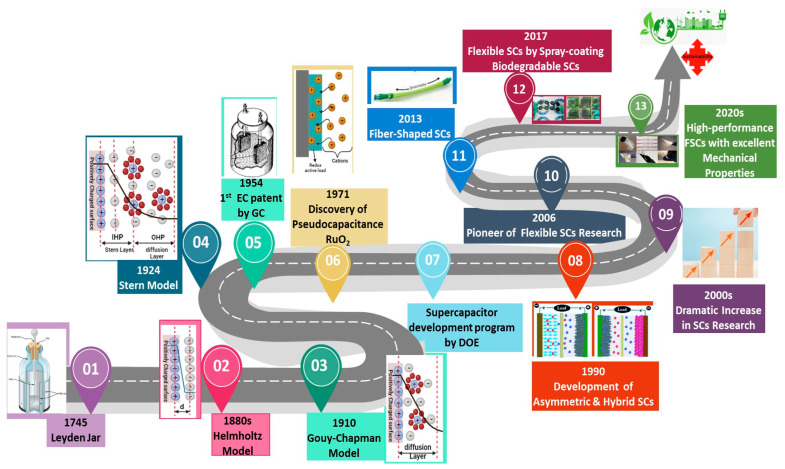
Development timeline of supercapacitors [35].

Helmholtz introduced the concept of electric double layer (EDL) in the 19th century (Figure 1). The Helmholtz model is based on the idea of particles with opposite charges forming layers at the interface between the electrode and the electrolyte, which are separated by atomic distances, just like in the case of regular capacitors. This was later improved by Gouy and Chapman (Figure 1), who introduced the concept of a diffuse layer, in which the ions surrounding the charged surface are not strongly bound to it but instead create a diffuse interface. The kinetic energy of the ions in the electrolyte partially affects the thickness of this diffuse interface [34,35].

Based on the earlier concepts, Stern presented a model in which the particles were divided into inner region (compact or Stern layer) and diffuse layer (Figure 1). Based on this theory, the size of ions is limited, restricting their access to the surface. The Stern layer is formed by ions adsorbed onto the surface, which form two planes: one consisting of specifically adsorbed ions (the inner Helmholtz plane, or IHP) and the other consisting of non-specifically adsorbed counterions (the outer Helmholtz plane, or OHP). This diffusion layer is defined by the Gouy–Chapman model where the diffusion layer is formed by the kinetic energy of the counterions and depends on the thickness of the layer [32,36,37]. The first patent for storing charges in EDL was issued in 1957 [38]. The same patent detailed the formation of the double layer of charges at the interface of the electrolyte and the solid material. During the 1960s, Standard Oil Company (Cleveland, OH, USA) committed itself to the development of this technology and made considerable progress; however, the technology’s subsequent development was halted due to a lack of sales. The Nippon Electric Company (NEC) in Tokyo, Japan, eventually acquired the technology in the 1970s [39]. The company began manufacturing low-power devices under the brand name “Supercapacitor” for memory storage [40]. Subsequently, this technology was adopted by other manufacturers, who launched their own branded versions, such as the “Panasonic Gold Capacitor” developed by the Matsushita Electric Industrial Company (Kadoma, Japan) in 1978 and “Dynacap” by ELNA Co. Ltd. (Yokohama, Japan) in 1987 [41,42]. The extensive fundamental work on ruthenium(IV) oxide (RuO_2_) in 1971 created a new class of electrochemical capacitors called pseudocapacitors. The newly discovered pseudocapacitance has enabled these devices to store greater amounts of charge [43]. In 1982, the Pinnacle Research Institute (Fort Lauderdale, FL, USA) developed the very first high-performance double-layer capacitor, which was marketed under the brand name “Ultracapacitor”. The US Department of Energy later pursued the development of hybrid electric vehicles using this technology, and in 1992, Maxwell Laboratories Inc. (San Diego, CA, USA) took over the development and began manufacturing a wide range of supercapacitors, including asymmetric supercapacitors, electric double-layer capacitors (EDLCs) and pseudocapacitors [44,45,46]. Nowadays, an increasing number of companies are involved in the manufacture of supercapacitors (Table 2), including Nippon Chemi-con Corporation (Tokyo, Japan), KEMET Electronics Corporation, a subsidiary of Yageo Corporation (Fort Lauderdale, FL, USA), CAP-XX Ltd. (Sydney, Australia), Ness Capacitor Co. Ltd. (Yongin-si, Republic of Korea), etc.

**Figure 2:**
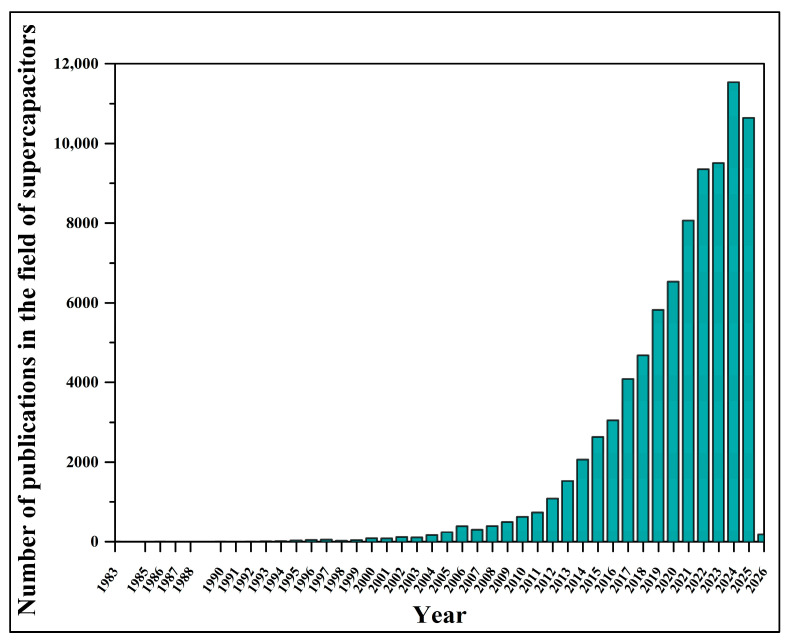
Timeline of publications in the field of supercapacitors from the beginning to the present day, based on Science Direct (https://www.sciencedirect.com, accessed on 24 August 2025).

The research in this field is constantly expanding (Figure 2) as scientists focus on advancing knowledge, bringing together the latest findings, and improving current understanding [40,48,49,50,51,52,53,54] towards a more sustainable future, evidenced by real-world applications such as supercapacitor-assisted buses for public transportation in urban areas of South Korea and China [55,56,57,58,59,60].

## 3. Principles of Supercapacitor Technology

This chapter reviews supercapacitors in more detail, discussing their classification and specifications. The operating principles of supercapacitors are basically like electrostatic capacitors.

### 3.1. Characteristics and Principles of Supercapacitors

**Figure 3:**
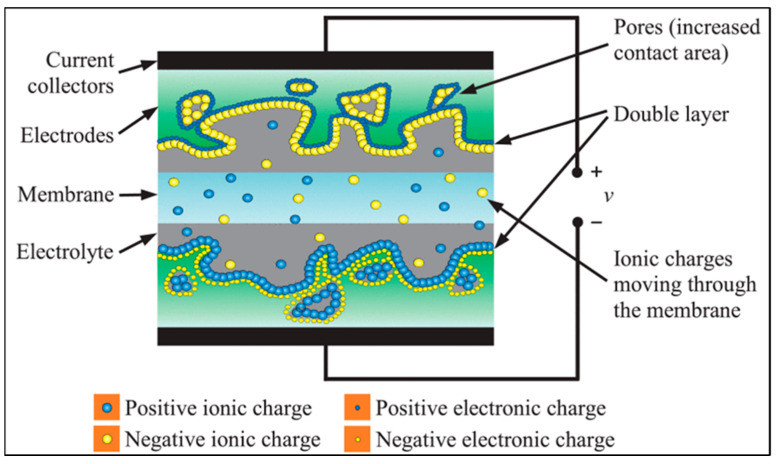
Scheme of a supercapacitor cell [65].

SCs integrate the strengths of batteries and conventional capacitors using the principles of electrostatics to regulate the efficient storage and release of energy [61]. Supercapacitors provide greater power density than batteries and greater energy density than conventional capacitors [62]. Supercapacitors (Figure 3) consist of an electrolyte, a separator, and two electrodes (anode and cathode). The electrolyte and electrodes generate an electric field which stores energy. Typically, the anode and cathode are porous materials separated by ion-permeable membranes [63]. In hybrid and asymmetric cells, the electrodes may differ from each other, whereas they are identical in symmetric cells. The main operating principle of supercapacitors is as follows [64]: when SC connects to the power supply during the charging process, the electrons are forced to migrate from the positive electrode to the negative electrode through this external circuit. This migration creates a potential difference across the SC; consequently, anions move toward the positive electrode and cations move toward the negative electrode in the electrolyte. To compensate for the imbalance of the external charge, electrostatic double layers are formed, in which energy is stored at the interfaces between the electrolyte and the electrodes. During the charging process, the voltage usually linearly rises across the supercapacitor, until it reaches its maximum nominal value. During discharge, the reverse of the process described above occurs when a device is connected to the SC: electrons migrate to the positive electrode through the external circuit, thereby transferring the energy stored in the SC to the connected device. Several multicharged ions can indeed dissociate, but in most cases, neutral molecules are dissociated in the electrolyte. The electrodes are essentially neutral, since the anions and cations migrate from the negative and positive electrodes, respectively. The voltage of the supercapacitor drops during discharge, typically in a linear sequence, until the energy is used up.

**Figure 4:**
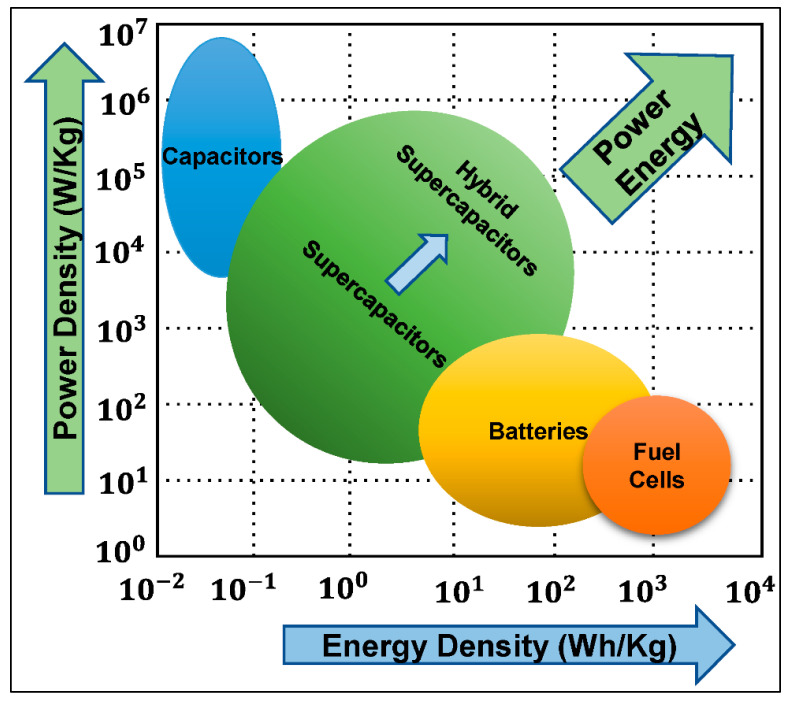
Ragone diagram: visual comparison of the power–energy density distribution of energy storage devices [66].

The comparison of different energy storage devices can be visualized with the Ragone diagram [56], which enables comparison by plotting power density (W/kg) on the *y*-axis and energy density (Wh/kg) on the *x*-axis (Figure 4).

As can be seen, traditional capacitors have the lowest capacity to store energy, yet they can discharge extremely fast, providing the highest power density. Momentary power output of batteries is limited, yet their ability to store considerable quantities of energy in a small volume is remarkable. Fuel cells can provide more energy density but require a series of chemical reactions to release energy. Supercapacitors fill the gap by bridging the divide between traditional capacitors, while offering an exceptional power density. Different subcategories of SCs exhibit further diversity in the energy–power space, as shown in Figure 4. Other relevant parameters must also be considered to achieve a complete understanding of advantages and limitations of these energy storage technologies besides the Ragone plot, including cycle lifetime and degradation, costs and resource extraction, toxicity, safety, etc. [35,54,67,68,69,70,71,72]. SCs offer considerable advantages in terms of lifetime, as the charge-storage mechanism allows them to operate between 100,000 and 1 million cycles. Charges are stored physically on the surface of the electrodes and do not involve irreversible chemical reactions, which is why SCs can greatly outperform batteries in terms of cycle life [73]. The lifespan of batteries is finite as swelling occurs in the active material during charge and discharge periods as a result of characteristic redox reactions. The volume of most SC electrodes does not change as their electrostatic storage is largely reversible. In addition, the mechanism of charge storage generates less heat than conventional rechargeable batteries, enabling safer and more robust operation [67].

### 3.2. Classification of Supercapacitors

Supercapacitors are classified by their manufacturing methods and structural design. They can have different shapes, like cylindrical, flat, or rectangular casings [74]. SCs can be classified into three major groups based on their energy storage mechanism: electric double-layer capacitors (EDLCs), pseudocapacitors (PCs), and hybrid capacitors. EDLCs and PCs differ from each other in terms of energy storage mechanism. In EDLCs, the double layer contains anions and cations which are accumulated at the interface between the electrodes and the electrolyte and electrostatically stored. The active electrode materials used in EDLCs are nanoporous materials with high specific surface area of more than 1000 m^2^/g. Such nanoporous materials are mainly carbon-based, as they are cheap, readily available, and easy to manufacture [75]. Carbon-based materials the most frequently used in EDLCs are carbon nanotubes (CNTs), activated carbon (AC), graphene, carbon aerogels, carbon foams, etc. [48,76,77]. EDLCs can store large number of charges, due to the high surface area of the electrode, resulting in higher capacitance values. The surface density depends upon the applied voltage; hence, the electrode capacitance changes with the electrode potential. The sole electrochemical reaction involves adsorption and desorption of ions on the electrode which makes this mechanism inherently fast energy storage [78]. The charge storage of PCs is based on faradaic process that involves the transfer of charge carriers electrostatically [79] by rapid redox reactions on or near the electrode surface when voltage is applied. The electrochemical behavior of PCs is similar to EDLCs, involves electron transfer, and changes the oxidation state of the electrode, which results in significantly higher specific capacitance than EDLCs. Three major types of pseudocapacitance can be classified as follows [80]: (i) redox pseudocapacitance, typically involving the electrochemical adsorption of ions near the electrode surface with faradaic charge transfer and occurring mainly in aqueous electrolytes, for example manganese(IV) dioxide (MnO_2_) and RuO_2_ [81]; (ii) intercalation pseudocapacitance, which happens by the intercalation of ions into the layers of redox-active materials together with faradaic charge transfer without the occurrence of a crystalline phase transition, similar to the intercalation of ions in the electrode of Li-ion batteries for which usually involves a phase transition [80] (including niobium pentoxide (Nb_2_O_5_) [82]; and (iii) adsorption pseudocapacitance, determined by single-layer adsorption, like platina electrode [83]. The rate of reaction, determined by surface coverage, intercalation, and surface redox, is nearly linearly dependent on potential (V) for all types [84,85]. The intercalation pseudocapacitance is one of the most promising types, which stores charge in the electrode material through rapid intercalation and deintercalation of ions, thereby overcoming the gap between conventional batteries and supercapacitors [86,87]. Conducting polymers and transition metal oxides can also be applied as electrode materials, combining the pseudocapacitive and electrostatic charge-storage mechanisms and enhancing energy density and the performance of SCs [54,88,89,90,91,92]. Conductive polymer-based SCs provide high capacitance and low internal resistance at lower production cost than carbon-based EDLCs [93]. One of their disadvantages is that they maintain a lower power density and shorter life cycle because of their poor mechanical stability as the electrodes swelling and shrinking during the redox reactions [40,84,94]. Hybrid capacitors integrate the energy storage capacity of PCs or battery-like electrodes and the energy delivery capacity of EDLC electrodes in a single cell. Hybrid capacitors combine both polarizable (e.g., carbon-based) and non-polarizable (e.g., metal or conductive polymer) electrodes. They can maintain high energy storage capacity through a combination of Faradaic and non-Faradaic processes derived from each electrode [95,96,97,98]. The result of combining both electrode types leads to the suppression of the limiting qualities of each electrode, thereby enabling the use of higher working potentials and exhibiting a higher specific capacity (generally two to three times higher) compared to EDLCs and pseudocapacitors. This enables the development of low-cost SCs with high electrical conductivity, high mechanical robustness, and high chemical resistance. There are three additional classes of hybrid capacitors: asymmetric and battery type and composite hybrid SCs [96,98,99].

### 3.3. Characteristics of Supercapacitors

The three fundamental parameters required for effective characterization of the energy storage capacity and performance of SCs: total capacity (C_T_), equivalent series resistance (R_ES_), and operating voltage (V_O_). Additional factors are also crucial in scientific research for the development of new electrode materials and innovative cell designs, including energy and power density, stable lifetime, operating voltage range, etc. To characterize the supercapacitive behavior of SCs, typically three techniques are used as follows: cyclic voltammetry, galvanostatic charge–discharge, and electrochemical impedance spectroscopy. The primary purpose of these techniques is to evaluate the electrochemical characteristics of energy storage systems from different perspectives. In general, parameters are measured as follows: current, time, and voltage. The other parameters can be calculated using these data [54].

Cyclic voltammetry (CV) can analyze the electrochemical behavior of electrode materials. If the used voltage is greater than the estimated voltage, the current can be checked with the Nernst equation [100]. During CV analysis, electrochemical behavior occurs during both scans (forward and backward), which can be distinguished from pseudocapacitive behavior based on the observed peaks and the general profile of the EDLC behavior of the electrode material [101]. The CV technique examines the electrochemical behavior of the electrode material in a three-electrode configuration with reference and counter electrodes. The main limitation of CV is that it overlooks thermodynamic aspects and only considers the kinetic properties of the material [102,103].

**Figure 5:**
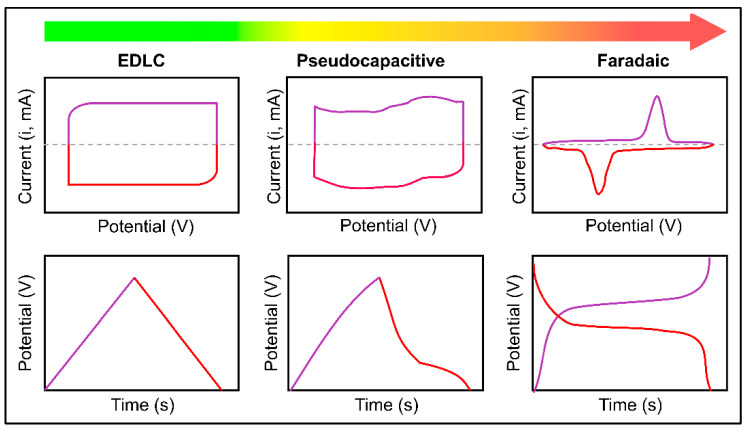
Schematic illustration of cyclic voltammograms and corresponding charge–discharge curves of EDLC, pseudocapacitor, and Faradaic materials [107] (Open Access).

The galvanostatic charge and discharge (GCD) technique analyzes the changes in potential over time. During the measurement, the electrode is charged and discharged at a specific point in time according to the properties of the material type (electrical activity, specific surface area, etc.) [104,105]. In particular, energy balance, power density, specific capacity, and stability are calculated from data on the potential window and charge and discharge times. The general shape of the charge–discharge diagram (Figure 5) shows the behavior differences of EDLC and pseudocapacitance based on the shape of the shoulders induced by Faraday currents [106,107].

Electrochemical impedance spectroscopy (EIS) is used to validate the parameters relevant for SCs. The data gained from EIS analysis is processed using appropriate software, such as ZView^®^ (Scribner Associates, Southern Pines, NC, USA), to determine the equivalent circuit required for analysis. The equivalent circuit features illustrate the overall properties of SCs, including equivalent series resistance, non-ideal behavior, and charge transfer capacitance [108].

Due to the different mechanisms of these measuring techniques, the results may seem contradictory; hence, in addition to the results, it is vital to indicate which parameters were used [54,109,110,111,112,113].

## 4. Carbon-Based Electrode Materials for Supercapacitors

The functional characteristics of SCs depend on the choice of materials used as electrolyte, electrode, separator, and collector. Nearly all characteristics of the structure and charge-storage mechanism of supercapacitors rely on these materials. Numerous studies have been conducted to enhance energy density, power density, lifetime, and voltage properties.

**Figure 6:**
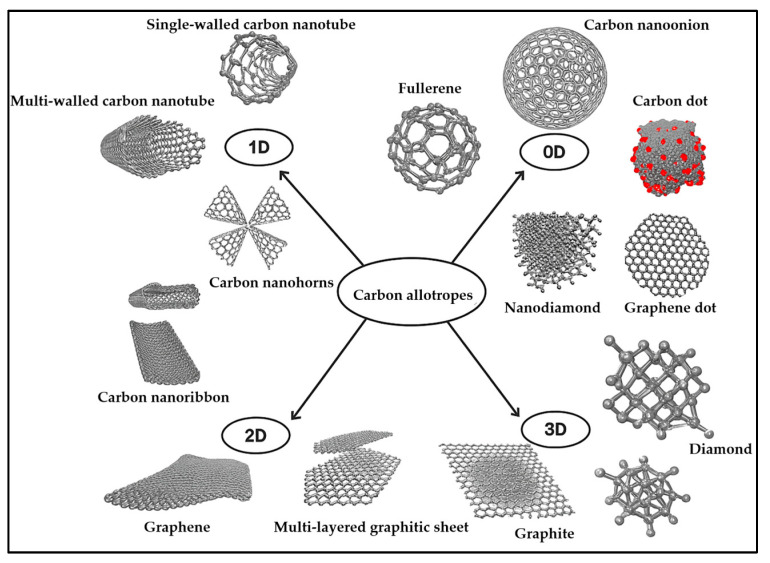
Schematic illustration of carbon allotropes; reproduced ref. [117] (Open Access).

The charging and storage capabilities of SCs are strongly dependent on the used electrode material; therefore, the development of SCs focuses on novel, enhanced-performance electrode materials. Ideally, electrodes should have excellent electrical conductivity, adequate electrochemical and thermal resistance, a high surface area for electrochemical activity, and good wettability by the electrolyte. Recyclability and cost-effectiveness are also important considerations [28,37,114,115]. The electrochemical performance of electrode materials depends on various parameters, including morphology, pore structure, and specific surface area. Carbon-based materials (Figure 6) have been long exploited as electrodes in energy storage, for example as electrically conductive additives, active material supports, electron transfer catalysts, intercalation supports, current collector substrates, and tools for controlling heat transfer, porosity, surface area, and capacitance [51,116,117].

The advantages of carbon as a supercapacitor electrode material stem from its unique combination of chemical and physical properties, in particular, high conductivity, large surface area (∼1 to >2000 m^2^/g), high resistance to corrosion, temperature stability, controlled pore structure, fabricability and compatibility in composite materials, and comparatively modest cost [13]. Thus, in the following, carbon-based electrodes will be presented, with an emphasis on carbon nanotube-based electrodes, a comparison of the specific capacity, energy and power density, retention, and cycle life properties of these materials is shown at the end of Section 4 (Table 3).

Activated carbon (AC) is a promising electrode material in energy storage systems. ACs are synthesized using both chemical (low temperature, in the presence of potassium hydroxide (KOH)) and physical (high temperature, in atmospheres of carbon dioxide) methods [118,119,120]. Activated carbon is usually made from carbon-rich materials [121]. The two most important sources of AC production are agricultural waste or biomass rich in carbon and lignocellulose materials. The raw materials used to produce commercially available activated carbon, such as petroleum residues, lignite, coal, and peat, are not cost-effective and are available in limited quantities [122]. The production of low-cost activated carbon from agricultural by-products such as grains, corn cobs, hemp hurd sticks, almond shells, and bamboo has been reported [122,123,124,125]. AC electrodes offer optimal performance in both aqueous and organic electrolytes, as these organic materials form small pores that are not involved in the electrochemical mechanism [126,127].

Carbon nano-onions (CNOs) are carbon skins with a spherical morphology, discovered by accident in transmission electron microscopy (TEM) image in 1980 by Iijima during his research on carbon black [128]. The sp^2^ network structure of CNOs allows rapid charge transfer and has been subsequently used as EDLC electrode material in the manufacture of SCs [129]. It has been found that, compared to other EDLC materials, there is no temperature limit to their energy storage performance, enabling their application in a broader temperature range [130,131]. CNOs have been produced by various synthesis methods, including annealing and arc discharge, that enabled industrial-scale production [132,133]. They are extensively applied in industry due to their low synthesis costs [134,135]. The energy density of CNOs can be up to 10 Wh/kg as a result of activation, and the specific capacity of CNOs is effectively increased. The activation of CNOs, on the other hand, might cause quality loss in these materials. Using CNOs as anodes and other pseudocapacitive materials as cathodes in asymmetric SCs can ultimately yield high-power, high-energy-density, and efficient energy storage solutions [129,136,137].

Graphene is a honeycomb-like single-layer structure of sp^2^-bonded carbon atoms. The structure of graphene consists of carbon atoms connected by covalent bonds to three neighboring carbon atoms and has unique electrical and thermal properties due to its single free electron [138]. As a 2D material, graphene has recently attracted considerable attention for use as a single-atom-thick electrode material in supercapacitor systems. Graphene-based electrode materials exhibit high specific surface area, leading to high electrical conductivity properties, chemical resistance, long cycle life, a narrow diffusion distance due to their thin structure, and great accessibility to functional groups. Based on these considerations, the extensive research and application of graphene as an integral part of developing energy storage devices is fully validated [139,140].

**Figure 7:**
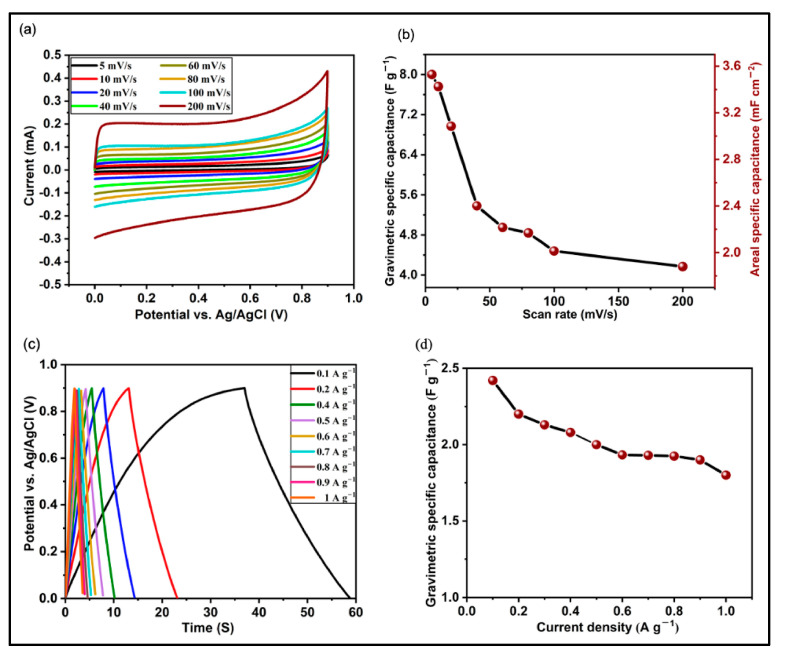
(**a**) CV curves of VACNT-based electrodes with different scan rates, (**b**) specific capacitance as a function of scan rate, (**c**) GCD curves at different current densities, and (**d**) specific capacitance as a function of current density [154] (Open Access).

Carbon nanotubes (CNTs) with a 1D structure, formed by the hybridization of sp^2^ carbon atoms, were discovered by Iijima during the TEM characterization of fullerenes synthesized via arc discharge technique in 1991 [141]. From the perspective of physical and chemical properties, CNTs have outstanding tensile strength, good elasticity, high electrical conductivity (106–107 S/m), high thermal conductivity (~2800–6000 W/m × K), and a variety of other advanced features [142,143,144,145,146,147,148,149,150]. These excellent properties have enabled the widespread use of CNTs in many scientific fields and practical applications, including supercapacitor energy storage systems. Based on the number of wall layers, CNTs can be classified into four categories: single-walled carbon nanotubes (SWCNTs), double-walled carbon nanotubes (DWCNTs), multi-walled carbon nanotubes (MWCNTs), and a special subtype of vertically aligned carbon nanotubes (VACNTs or CNT forests) [151,152,153]. Theoretically, CNTs can have relatively high specific surface area (1100–1500 m^2^/g) and high conductive properties, that make them promising candidates for energy storage devices. In a new study, CNT forests were grown on copper foil using the plasma-enhanced chemical vapor deposition technique [154]. Electrochemical tests confirmed (Figure 7) that the gravimetric specific capacitance was 8 F/g and the areal specific capacitance was 3.5 mF/cm^−2^ at a scan rate of 5 mV/s. The electrode fabricated from these CNT forests demonstrated outstanding rate capability and retained 92% of its initial capacity after 3000 cycles.

However, CNTs naturally aggregate easily due to Van der Waals interactions and weak dispersion capabilities, resulting a dramatic decrease in specific surface area (up to 50 m^2^/g), and eventually electrochemical performance. To solve these problems, CNT production methods and chemical functionalization/modification have been developed [155]. In the beginning, CNTs were manufactured using arc discharge. This method was well known and was used to synthesize carbon fibers and carbon filaments. Later, other techniques were tried to synthesize CNTs: laser ablation or chemical vapor deposition (CVD). In fact, these three are the main synthesis techniques for CNTs. Some attempts have been made to prepare carbon nanotubes using other methods, but these have not been promising alternatives. This may be due to the expensive reaction equipment, the price of used materials, or the extreme reaction environment, including high pressure or liquid nitrogen temperature [156]. Many studies have attempted to enhance the quality and the quantity of CNTs by optimizing the synthesis process. As a result, some types of CVD method were discovered as follows: microwave-enhanced, plasma-enhanced, radiofrequency-enhanced CVD, etc. [157]. Unlike conventional CNTs, vertically aligned carbon nanotubes can only be prepared efficiently using catalyst-supported chemical vapor deposition (CCVD) technology [158,159]. VACNT structures can be grown on different substrates, mainly silicon; however, there are many efforts to synthetize them on conductive metal substrates, including titanium (Ti), aluminum (Al), and stainless steel (SS) [160,161,162,163,164,165].

For integrated application in SCs, it is preferred for VACNTs to grow directly on conductive substrates, particularly metal substrates. In this way, the manufactured electrode materials can improve electrical performance by facilitating enhanced electrical contact between VACNTs and metallic current collectors and simplifying the electrode assembly process by eliminating the need for conductive binders and dopants in the electrode materials [166]. The synthesis of VACNTs includes catalytic decomposition of carbon compounds, mainly hydrocarbons, at elevated temperatures using metal catalyst nanoparticles supported on an oxide buffer layer, which are deposited on the substrate surface. Previous studies have revealed that the metal catalysts, buffer layer, and substrate have great impact on the growth of the VACNTs [167,168,169,170,171,172]. Transition metals, including iron, cobalt, and nickel, are extensively used as catalysts in the CVD method. Catalyst layers can be deposited in the form of single-component, bi-component or multi-component layer on the substrate by various thin layer deposition techniques, including physical vapor deposition, magnetron sputtering, atomic layer deposition, dip coating, thermal evaporation, pulsed laser deposition, etc. [173,174,175,176]. The properties of the synthesized VACNTs often do not meet the desired requirements, therefore, depending on the intended application and the required properties, different functionalization of VACNTs are possible: uniformly along the entire length of the nanotubes, or selectively at the CNT tips and CNT sidewalls [177,178]. Doping CNTs with heteroatoms like nitrogen, boron, and phosphorus is an effective approach to improve the specific capacitance of CNTs. Heteroatoms on the surface of CNTs can result an increase in conductivity and thus improve the specific capacitance of the structure, for example nitrogen-doped CNTs have approximately twice the specific capacity of pure CNTs [179,180,181,182].

**Figure 8:**
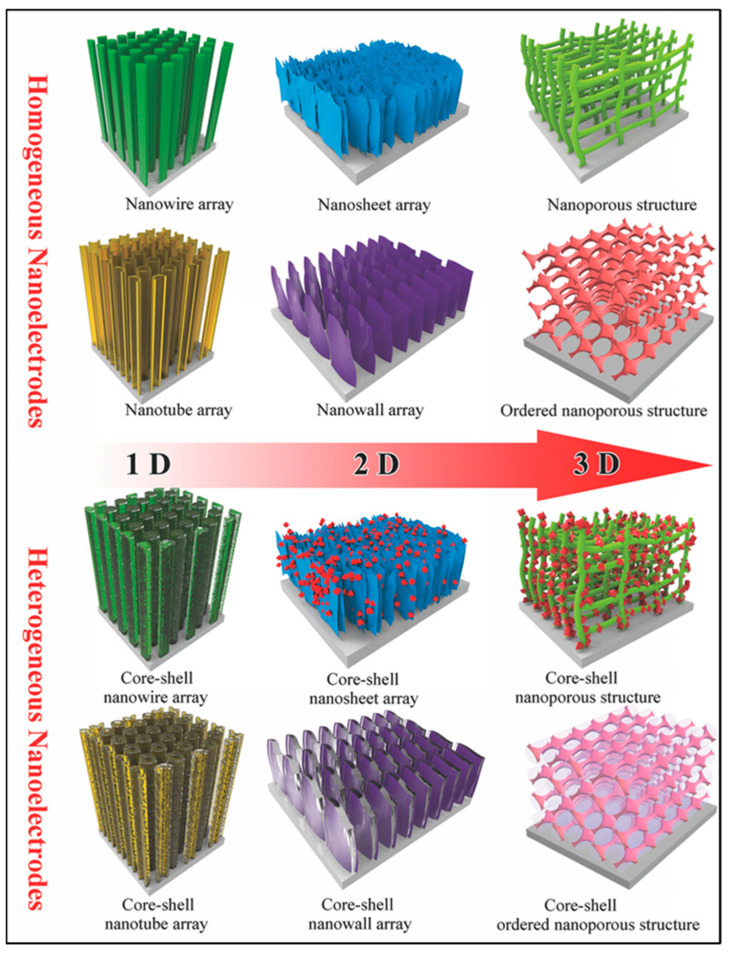
Scheme of homogeneous and heterogeneous nanoelectrodes used for SCs [184].

CNTs and other carbon-based nanomaterials have been combined with transition metal oxides to form composite materials. It is generally believed that the real nanoelectrodes for supercapacitors primarily refer to electroactive materials consisting of various independent nano-arrays grown on conductive substrates. To produce supercapacitor nanoelectrodes, the most used method is to directly manufacture electroactive materials in either one-dimensional (1D) nanomatrices (nanowires, nanorods, and nanotubes) or two-dimensional (2D) nanomatrices (nanosheets and nanowalls) or three-dimensional (3D) nanoporous architectures (Figure 8) [183,184,185,186,187]. Thus, the production of VACNTs on conductive substrates is a promising area.

The development of 1D, 2D, and 3D carbon-based nanostructures as electrode materials, including CNO and CNT-based arrays and graphene-based architectures, can create hierarchical porous channels due to structural interconnections. These structures also exhibit higher electrical conductivity and better structural mechanical stability, which can improve the performance metrics of supercapacitors, such as higher energy density, power density, and longer lifespan. These materials can improve the transport of ions and electrons, while the porous structure provides a large surface area for charge storage and allows for the deposition of other high-performance pseudoactive materials, such as metal oxides or conductive polymers. This can enable the development of hybrid electrodes that bridge the disadvantages of carbon-based nanostructures and pseudoactive materials and synchronize their advantageous properties [188,189,190].

For the assembly of nanocomposites based on carbon nanotubes and transition metal oxides, various methods have been used, including atomic layer deposition, which is a time- and cost-consuming technology [191]. Recently, other methods, including hydrothermal, electrochemical deposition, anodization, CVD, electrospinning, etc., techniques, have been used for the synthesis of these nanocomposites [183,192,193,194,195]. There are several types of hydrothermal processes based on different mechanisms, including solvothermal, autogenous, molten salt, and microwave-assisted [196,197,198,199]. Each of these techniques is suitable for the fabrication of nanocomposites.

MWCNT electrodes coated with nickel(II) oxide (NiO)–MnO_2_ are obtained by a straightforward chemical precipitation process with a defined capacity of 193.5 F/g (at a scan rate of 5 mV/s) in 6 mol/dm^3^ KOH electrolyte. The observed GCD curves exhibited symmetrical triangles, suggesting superior EDLC behavior and reversible charging/discharging performances [200]. Gold coatings were deposited on VACNTs using a direct current (DC) magnetron sputtering method, and the porous gold (Au) deposit was anchored to VACNTs with a large apparent surface area. The porous Au/VACNT nanoelectrodes have good surface capacitance compared to most modern gold-based electrodes (25.6 mF/cm^2^ at 5 mV/s) and excellent cycle stability (90% retention after 10,000 cycles) in 0.5 mol/dm^3^ sulfuric acid (H_2_SO_4_) aqueous solution [201]. Poly(3-methylthiophen) (P3MT) was deposited via potentiostatic pulse deposition on VACNTs grown directly on aluminum foil. VACNTs with high density (2.1011 VACNT/cm^2^) and anisotropic properties had a large specific surface area (340 m^2^/g) and served as template electrodes for the formation of the P3MT coating nanostructure. The deposited P3MT layer was up to 70% deposited inside the mat on the VACNTs, resulting in a specific capacity of 170 F/g. The calculated areal capacity was 380 mF/cm^2^, and the volumetric capacity was 76 F/cm^2^. In addition, the nanostructured electrodes showed great stability and capacity retention after 19,000 cycles. Asymmetric electrochemical capacitors were finally assembled, with a specific energy of 52 Wh/kg (14 Wh/L) and a specific power of 10 kW/kg [202]. A composite electrode with homogeneous vanadium(V) oxide (V_2_O_5_) nanoparticles were prepared via supercritical carbon dioxide (CO_2_) impregnation followed by heating and used as a binder-free negative electrode in aqueous asymmetric supercapacitors. The V_2_O_5_/VACNT composite electrode exhibited ideal specific capacity (284 F/g) in the potential range between −1.1 and 0 V at 2 A/g compared to saturated calomel electrode, as well as outstanding cycle stability in aqueous sodium sulfate (Na_2_SO_4_) solution. Asymmetric supercapacitors provided a high energy density of 32.3 Wh/kg at a power density of 118 W/kg and exhibited satisfactory cycle life with 76% capacity retention after 5000 cycles [203]. CNT composite nanoelectrodes decorated with Fe_3_O_4_ nanoparticles were prepared using a microwave solvothermal method. The prepared Fe_3_O_4_/CNT composite exhibited a reversible capacity of 187.1 F/g at 1 A/g, superior rate capability by maintaining 61.6% of 10 A/g (vs. 1 A/g), and 80.2% cycle stability after 1000 cycles at 1 A/g [204]. The performance and cycle stability of these composite electrodes can be significantly improved through increased conductivity [205,206].

Among the carbon-based electrode materials for SCs, porous carbon materials with a 3D structure also offer excellent electrochemical performance as cathode materials [185,186,187]. These 3D structured materials are transformed into complex polymer superstructures with characteristic flower-like morphology and structure through the controlled growth of oligomers. The resulting hierarchical porous carbon superstructures exhibited a significant surface area (2824 m^2^/g) providing alternative and efficient charging routes. It is noteworthy that in the hybrid system of these carbon superstructures with zinc ions, the synergistic combination of dual ion storage, ion-accessible pore structures, and endogenous zincophilic sites led to a superior performance, which included a remarkable energy density of 112.1 W/kg and 160.8 Wh/kg, exceptional cycle stability of 200,000 cycles at 20 A/g, and a high specific capacity of 262.8 mAh/g at 0.2 A/g [185].

**Table 3:** Electrochemical performance characteristics of carbon-based materials for supercapacitors.

Type of Electrode	Sample Name	Capacitance (F/g)	Energy Density (Wh/kg)	Power Density (kW/kg)	Retention/Cycles (%)	Ref.
Graphene	rGO	585.44	81.31	62.64	94.14/5000	[207]
	NiO@srGO/CNT	1605.82	N/A	N/A	71.56/10,000	[197]
	NMGO//MWCNT	90	28	0.75	88/6000	[208]
	4NG	405	68.1	558.5	87.7/5000	[209]
Activated Carbon	RPC	56	44	0.564	N/A	[210]
	HAC-WS	225	72.2	1.547	88/2500	[211]
	hCNC-5.0	281	153	1.000	93/100,000	[212]
Carbon Nanotubes	CNT Am-241	489.6	68	9.992	98.5/5000	[213]
	VACNT	8	0.20	0.450	92/3000	[154]
	Fe_3_O_4_/CNT	187.1	N/A	N/A	80.2/1000	[204]
	V_2_O_5_/VACNT	284	32.3	0.118	76/5000	[203]
	P3MT/VACNT	170	52	10	95/19,000	[202]
	PC-CNT	248	8.42	0.250	97.3/3000	[214]
	SWCNT/TiO_2_	144	20	10.000	95/50,000	[215]
	CNT-SC	375.4	75.1	N/A	93.1/100	[216]
	CNT/TiNiW-SC	549.1	336.7	N/A	95.4/100	
	CNT-NF	250.5	68.19	27.994	92.42/10,000	[217]
	G/CNT-SP	500.16	69.46	N/A	87/1000	[218]
	MnO_2_@CNT	219	N/A	N/A	88/7000	[219]
Carbon Nano-onions	Co_3_O_4_/CNO	402.35	N/A	N/A	76/9000	[220]
	PEDOT-MoO_3_@CNO	428	N/A	N/A	76/6000	[131]
	RFM-CNO-C	160	5	0.43	97/3000	[221]

In previous research, various electrode fabrication techniques (such as screen printing, etching, pressing, lamination, deposition, coating, and direct synthesis on the current collector), electrolyte systems (such as aqueous and organic), and substrates (conductive and insulating) have been used [222,223,224,225,226,227]. The obtained properties can vary significantly, thus making it difficult to directly compare the electrochemical performance of active materials. Furthermore, few studies have addressed the use of spin coating and inkjet printing techniques in electrode fabrication, as increasing the loading of active materials generally improves the overall capacity, and there has been limited interest in extremely lightweight energy storage systems [228,229,230,231]. This suggests that the manufacturing technology itself can have a significant impact on the properties of the device for the following reasons: (i) minor changes in the manufacturing method can result in various microstructures, which affects the properties of these electrode materials, and (ii) the electrochemical performance of SCs depends on both the width of the electrodes and the interspace between the electrode pairs. The accuracy and precision of the above parameters are also influenced by the fabrication methods, which cause differences in electrochemical performance (see Table 3).

## 5. Conclusions and Outlook

Supercapacitors offer a promising alternative to overcome long-term energy storage problems. They are reliable and best suited for high-power transfer applications, but it is advisable to combine them with batteries. SCs can be manufactured using a variety of methods and materials. This study reviews the fundamentals of supercapacitors, their structure, properties, and the various types of carbon-based electrodes used in their construction. The high interest in carbon-based nanomaterials is also due to their high conductivity, morphological versatility, fine-tunable porous structure, controllable surface functions, natural abundance of raw materials, and low cost. VACNTs can not only provide a well-oriented “highway” for electron conduction, but also promote rapid ion transport due to the regular, small distances between CNTs and their alignment. High surface area of aligned CNTs on conductive substrates and introducing coating of transition metal oxides (NiO, V_2_O_5_, MnO_2_, ZnO, etc.) in order to increase the energy density are in current demand and meet the requirements to produce low cost, satisfactory cycle lifetime stability and high-energy-density supercapacitors. To achieve the full potential application of these devices, the performance and reproducibility of electrodes must be enhanced by further designing and developing these nanostructures. Therefore, studies focusing on the development of nanoscale materials that improve the capacitive performance of supercapacitors while maintaining high cycle life and dynamic reversibility are of particular importance. The hybridization of carbon-based nanostructures and metal oxides to form composite materials is recommended and is expected to yield significant success and coordinated efforts. Another important aspect is the possibility of integrating batteries and supercapacitors, as the strengths of one compensate for the weaknesses of the other. Nevertheless, future studies should focus on the following research directions: (i) developing new materials and designs that can significantly increase the energy density of supercapacitors, (ii) developing hybrid systems that combine the advantages of supercapacitors and batteries, thereby providing greater energy density and longer life cycles, (iii) developing environmentally friendly and sustainable materials, and (iv) novel materials and designs need to be developed to improve the reliability and lifespan (charge/discharge cycle) of SCs. Key challenges include bridging the gap between laboratory performance and commercial viability, and transitioning to more environmentally friendly processing methods. In the future, integrating advances in electrodes and engineering design will be essential to harnessing the full potential of SCs in modern energy systems.

## Figures and Tables

**Table 2:** Commercially available SCs (original data from ref. [47] (Open Access) and updated based on available information on the website of each company on 28 October 2025).

Name of Company	Country of Origin	Rated Voltage (V)	Rated Capacitance (F)	Equivalent Series Resistance (mΩ)	Specific Energy (Wh/kg)	Specific Power (kW/kg)
Asahi Glass	Japan	2.70	55–1350	2.50	10–100	0.1–2.0
BatScap	France	2.70–3.0	325–3000			
CapTop	Italy	2.70–3.0	1–5000	0.11–3.96	4904	5–40
CAP XX	Australia	2.5–3.0	0.035–3000	24–1300	N/A	N/A
Ioxus	USA	2.85–3.0	1152–3000	0.20–0.32	5–30	7.8–26
Kemet	Taiwan	2.7	0.047–2000	4–100	N/A	N/A
Maxwell	Republic of Korea	2.3–3.0	3–3400	0.13–62	4–7	18
Nesscap Energy	Republic of Korea	2.7	3–100	12–55	N/A	N/A
Nichicon	Malaysia	2.7	1–4000	18–80	5–10	
Panasonic	Japan	2.1–2.7	3.3–100	0.08–30	5	0.29
Skeleton	Estonia	2.85–3.0	330–5000	0.47–1.0	5.82–11.1	19.9–80

## Data Availability

No new data were created or analyzed in this study. Data sharing is not applicable to this article.

## Abbreviations

1D	One-Dimensional
2D	Two-Dimensional
3D	Three-Dimensional
AC	Activated Carbon
Al	Aluminum
Au	Gold
CCVD	Catalyst-Supported Chemical Vapor Deposition
CNO	Carbon Nano-onion
CNT	Carbon Nanotube
CO_2_	Carbon Dioxide
C_T_	Total Capacity
CV	Cyclic Voltammetry
CVD	Chemical Vapor Deposition
DC	Direct Current
DWCNT	Double-Walled Carbon Nanotube
EDL	Electric Double Layer
EDLC	Electric Double-Layer Capacitor
EIS	Electrochemical Impedance Spectroscopy
GCD	Galvanostatic Charge–Discharge
GO	Graphene Oxide
HAC	Hierarchical Activated Carbon
hCNC	Hierarchical Carbon Nanocages
H_2_SO_4_	Sulfuric Acid
IHP	Inner Helmholtz Plane
KOH	Potassium Hydroxide
MnO_2_	Manganese(IV) Dioxide
MWCNT	Multi-Walled Carbon Nanotube
Na_2_SO_4_	Sodium Sulfate
Nb_2_O_5_	Niobium Pentoxide
NEC	Nippon Electric Company
NG	Nitrogen Doped Graphene
NiO	Nickel(II) Oxide
OHP	Outer Helmholtz Plane
P3MT	Poly(3-Methylthiophene)
PC	Pseudocapacitor
R_ES_	Equivalent Series Resistance
rGO	Reduced Graphene Oxide
RPC	Rose Petal-Derived Porous Carbons
RuO_2_	Ruthenium(IV) Oxide
SC	Supercapacitor
SS	Stainless Steal
SWCNT	Single-Walled Carbon Nanotube
TEM	Transmission Electron Microscopy
Ti	Titanium
V_2_O_5_	Vanadium(V) Oxide
VACNT	Vertically Aligned Carbon Nanotube
V_o_	Operating Voltage
